# Action ability modulates time-to-collision judgments

**DOI:** 10.1007/s00221-017-5008-2

**Published:** 2017-06-12

**Authors:** Eleonora Vagnoni, Vasiliki Andreanidou, Stella F. Lourenco, Matthew R. Longo

**Affiliations:** 1 Department of Psychological Sciences, Birkbeck, University of London, London, UK; 2 Department of Clinical Educational and Health Psychology, University College London, London, UK; 3 Department of Psychology, Emory University, Atlanta, USA

**Keywords:** Time-to-collision, Looming, Action ability, Peripersonal space representation, Motor ability, Emotion

## Abstract

Time-to-collision (TTC) underestimation has been interpreted as an adaptive response that allows observers to have more time to engage in a defensive behaviour. This bias seems, therefore, strongly linked to action preparation. There is evidence that the observer’s physical fitness modulates the underestimation effect so that people who need more time to react (i.e. those with less physical fitness) show a stronger underestimation effect. Here we investigated whether this bias is influenced by the momentary action capability of the observers. In the first experiment, participants estimated the time-to-collision of threatening or non-threatening stimuli while being mildly immobilized (with a chin rest) or while standing freely. Having reduced the possibility of movement led participants to show more underestimation of the approaching stimuli. However, this effect was not stronger for threatening relative to non-threatening stimuli. The effect of the action capability found in the first experiment could be interpreted as an expansion of peripersonal space (PPS). In the second experiment, we thus investigated the generality of this effect using an established paradigm to measure the size of peripersonal space. Participants bisected lines from different distances while in the chin rest or standing freely. The results replicated the classic left-to-right gradient in lateral spatial attention with increasing viewing distance, but no effect of immobilization was found. The manipulation of the momentary action capability of the observers influenced the participants’ performance in the TTC task but not in the line bisection task. These results are discussed in relation to the different functions of PPS.

## Introduction

In vision, *looming* refers to a specific pattern of optical expansion of a surface or surface patch during direct approach towards a viewer (Gibson 1958). Looming stimuli evoke fear responses in crabs (Oliva et al. 2007), locusts (Gabbiani et al. 2002; Gray et al. 2010; Hatsopoulos et al. 1995; Jones and Gabbiani 2010; Rind 1996; Rind and Simmons 1992, 1997, 1999), goldfish (Preuss et al. 2006), frogs (Ishikane et al. 2005), pigeons (Frost and Sun 2004; Sun and Frost 1998; Wu et al. 2005; Xiao and Frost 2009; Xiao et al. 2006), monkeys (Schiff et al. 1962) and humans (Ball and Tronick 1971; Náñez 1988; Yonas et al. 1979; King et al. 1992). In monkeys, a network of brain areas has been identified in which multimodal neurons typically respond to objects touching, near, or looming toward the body surface (Graziano and Cooke 2006). This network represents the space around the body, also called peripersonal space (PPS) (Rizzolatti et al. 1997), and plays a role in the sensory guidance of movements toward objects (Gentilucci et al. 1988; Rizzolatti et al. 1988) as well as in reacting to or avoiding approaching objects (Cooke and Graziano 2003; Graziano et al. 2002). There is evidence coming from behavioural, neuropsychological and imaging studies in favour of a functionally similar PPS representation in humans (Halligan and Marshall 1991; Cowey et al. 1994; Spence et al. 2004; Holmes 2004; Brozzoli et al. 2011; Bremmer et al. 2001; Makin et al. 2007; Sereno and Huang 2014).

The optical expansion of an object on a direct collision course with a viewer, in theory, exactly specifies its time-to-collision (TTC) (Gibson 1958). However, a large body of research has shown that the arrival time of such looming stimuli is consistently underestimated (McLeod and Ross 1983; Schiff and Oldak 1990; Neuhoff 2001). The bias to underestimate the approach of looming stimuli may be an adaptation that provides a selective advantage (Neuhoff 1998, 2001). Indeed, underestimating TTC yields more time to engage in preparatory defensive behaviours. Although such a bias is, technically speaking, an error, the cost of a false positive (making preparatory actions too early) is far less than the cost of a false negative (making preparatory actions too late; Haselton and Nettle 2006). Following this logic, Neuhoff and colleagues have argued that perceiving and acting in response to looming stimuli depends not only on perceptual abilities, but also on the motor capabilities of the observer (Neuhoff et al. 2012). Indeed, the authors (Neuhoff et al. 2012) demonstrated how physical fitness modulates TTC judgments with listeners with poorer physical fitness showing a greater underestimation of the arrival time of looming sounds than listeners with better physical fitness.

A distinction can be made between moment-to-moment action capability (momentary action capability) and inherent action capability (stable action capability) (Kandula et al. 2016). Inherent action capability represents the set of motor skills and strategies the person possesses, whereas momentary action capability refers to the robustness of these skills to cope with the current task difficulty level (Witt 2011). It has been shown that inherent and momentary action capabilities interact to influence the location’s perception of an approaching ball (Kandula et al. 2016). Specifically, Kandula et al. (2016) showed that participants with low inherent action capability (non-video game players) underestimated the spatial location of an approaching ball only when their momentary action capabilities were low (e.g. when the task was difficult). This effect was not present in participants with high inherent action capability (video game players) who showed a more accurate spatial location perception independently from the task’s difficulty level.

Here we investigated whether moment-to-moment changes in the ability to act would similarly alter judged TTC. We reduced participants’ freedom to move by asking them to rest their chin in a chin rest. We hypothesized that having reduced freedom of movement would lead to an increased margin of safety, resulting in judgments of looming stimuli arriving sooner. Indeed, according to several authors, emotions, states or capabilities of the body can alter perception (Stefanucci et al. 2008; Stefanucci and Proffitt 2009; Proffitt et al. 1995). What we see in the world is influenced not only by optical and ocular-motor information, but also by one’s purposes, physiological state, and emotions (Proffitt 2006). Bhalla and Proffitt (1999), for example, showed that participants who wore a heavy backpack reported a hill as steeper than those who did not. These results have been contested and interpreted as effects of experimental demand characteristics (Dean et al. 2016; Durgin et al. 2009, 2012; Firestone 2013; Firestone and Scholl 2014). However, it has been shown that effort influences the peripersonal space representation and motor imagery using paradigms where the experimental hypotheses were, possibly, less transparent (Decety et al. 1989; Lourenco and Longo 2009). Other studies have shown that emotion influences perception and representation of space with the fear of heights being associated with distorted perception of vertical distance (Jackson 2009; Stefanucci and Proffitt 2009; Teachman et al. 2008), and claustrophobic fear associated with increased size of PPS representation (Lourenco et al. 2011; Hunley et al. 2017).

Although looming has been viewed as a simple optical effect, the semantic content of objects approaching our bodies and our individual differences related to fear modulates our responses. For example, participants underestimate the arrival time of threatening, relative to non-threatening, stimuli (Brendel et al. 2012; Vagnoni et al. 2012, 2015). Moreover, these effects are modulated by the specific fears of observers, with people more fearful of threatening stimuli underestimating more their arrival time (Vagnoni et al. 2012, 2015). This evidence is in line with and expands the view of the looming underestimation as an adaptive response (Neuhoff et al. 2012). Indeed, if it is true that observers ensure themselves with enough time to engage in a defensive behaviour if something is approaching their body this could be especially true if they fear the object that is approaching. In addition to manipulating action ability, we also manipulated the semantic content of the stimuli to test whether dangerous objects are perceived as arriving sooner when our body is mildly immobilized.

This study investigated the effects of mild immobilization in a chin rest on TTC judgments. In Experiment 1, participants made TTC judgments of threatening (snakes, spiders) and non-threatening (butterflies, rabbits) stimuli which expanded on a screen for 1 s at rates consistent with five actual TTCs. After each stimulus disappeared, participants were asked to imagine it continuing to approach at the same rate and to press a button when they judged that it would collide with them. In half of the blocks, participants stood with their chin resting in a chin rest, whereas in the other half of the blocks, they stood freely. We predicted that immobilization in the chin rest would lead participants to use a larger margin of safety around their body, and so to judge stimuli as arriving sooner. We were further interested in whether any such effect would be larger for threatening than for non-threatening stimuli.

The results of the first experiment showed stronger underestimation when participants had restricted ability to move suggesting an expansion of PPS. In the second experiment we wanted to test the generality of the effect using a different PPS paradigm. Previous findings have shown that when participants perform a line bisection task they show leftward bias in near space (*pseudoneglect*) and rightward shifts in bias with increasingly farther distances (e.g. Varnava et al. 2002; Longo and Lourenco 2006, 2007; Gamberini et al. 2008). The line bisection task has been used to investigate how PPS expands after tool use (Longo and Lourenco 2006; Gamberini et al. 2008; Seraglia et al. 2012), shrinks with the use of wrist weights (Lourenco and Longo 2009), and is modulated by claustrophobic fear (Lourenco et al. 2011). In the second experiment we used a different task to investigate the effect of restricted ability of movement on the PPS representation. The results of this experiment showed the classic leftward bias in near space but no effect of the manipulation. This lack of effect could be due to the fact that in the two experiments we tapped into different aspects of the peripersonal space representation.

## Experiment 1: Does reduced ability of movement influence time-to-collision judgments?

### Method

#### Participants

Thirty members of the Birkbeck community (20 female) between 20 and 58 years of age, mean age 31.8 years, participated for payment or course credit. Participants were generally right-handed as assessed by the Edinburgh Inventory (M: 78.2, range −100 to 100; 1 participant was left handed) (Oldfield 1971). Participants reported normal or corrected-to-normal vision. Procedures were approved by the local ethics committee.

An additional ten members of the Birkbeck community (6 female) between 20 and 49 years of age, mean age 26.2 years, completed an abbreviated version of the TTC task and a questionnaire about the experimental hypotheses for payment or course credit. Participants reported normal or corrected-to-normal vision.

#### Stimuli, design, and procedure

Stimuli were the same as used in our previous experiments (Vagnoni et al. 2012, 2015), namely 160 colour photographs collected from the internet, 40 from each of the four categories (snakes, spiders, butterflies, and rabbits). Images were cropped and resized using Adobe Photoshop CS5 (Adobe Systems, San Jose, CA). This resulted in images (400 pixels wide, 250 pixels high) in which the animal took up the entire image. Backgrounds from the original photographs were replaced with a homogenous grey colour (identical to the background of the experimental script).

Participants stood 60 cm from a screen (75 Hz refresh rate). Stimulus presentation and data collection were controlled by a custom MATLAB (Mathworks, Natick, MA) script using the Cogent Graphics toolbox (developed by John Romaya at the LON at the Wellcome Department of Imaging Neuroscience, University College London).

We encumbered the participants by having them rest their chin in a chin rest. In the “chin rest” condition, the participants stood in front of the screen resting their chin in a chin rest. In the “no chin rest” condition, they were simply standing in front of the monitor. All participants performed both conditions in a counterbalanced order.

To ensure that the participants maintained a constant distance from the screen and, more importantly, to control their position in the two different conditions they were asked to find a comfortable position of the head and to maintain it during the entire task without changing it between blocks. Moreover, we controlled the position of their feet by aligning them with strips of tape on the floor.

The monitor was positioned to be at the level of the participant’s head so that the stimuli were presented looming towards their face. On each trial, the stimulus increased in size across 75 frames (i.e. one second), consistent with one of five time-to-collisions (3.0, 3.5, 4.0, 4.5, and 5.0 s after the onset of the first frame). It is important to stress that the stimuli are perceived as approaching through their expansion. The stimuli, obviously, never moved on a horizontal plane, and the different time-to-collisions were set through a script that controlled the stimuli’s rate of expansion. The width of the stimulus on the first frame was either 400 or 500 pixels (for all the categories), which means that the size on the screen was either 10.6 or 13.3 cm (10° and 12.6° visual angle from 60 cm distance). The starting image size was manipulated so that actual time-to-collision was not perfectly correlated with the size of the image on the final frame. After the 75th frame, the image was replaced by a grey background.

There were a total of 160 trials divided into four blocks of 20 trials per condition (chin rest, no chin rest). Each block included one repetition of each combination of TTC (five levels) and stimulus category (four levels). The order of trials within each block was randomized. The 20 images from each category were randomly assigned to trial types and each image was used exactly twice for each participant. After the participant responded on each trial, the next trial began after a random inter-trial interval of 300–800 ms.

Fear ratings for each of the four categories were collected by modifying the Fear of Spiders Questionnaire (Szymanski and O’Donohue 1995). The 18 items on this questionnaire asked participants to indicate their agreement or disagreement with statements indicating fear or anxiety related to spiders. Example items included: “If I saw a spider now, I would feel very panicky” and “I now would do anything to try to avoid a spider”. The 18 statements were modified for each of the other stimulus categories by replacing the word “spider” with either “snake”, “butterfly”, or “rabbit”. Participants rated their agreement or disagreement with each statement using a 7-point Likert scale, where a score of +3 indicated strong agreement with the statement (i.e. high levels of fear) and −3 indicated strong disagreement (i.e. low levels of fear). The 72 items were presented in random order using a custom MATLAB script.

The participants also completed the Claustrophobia Questionnaire (CLQ; Rachman and Taylor 1993; Radomsky et al. 2001), a 26-item self-report questionnaire assessing trait claustrophobic fear. They had to indicate for each item how anxious they would feel in the described situations from 1 (not at all anxious) to 5 (extremely anxious). This questionnaire can be divided into two subscales, “fear of suffocation scale” (SS) and “fear of restriction scale” (RS). An example item of the first subscale includes “Having a bad cold and finding it difficult to breathe through your nose” while an example item of the second scale includes “Tied up with hands behind back for 15 min”.

### Results

Regarding the time-to-collision judgments, for each participant, *Z*-scores were calculated for time-to-collision judgments, separately for each level of actual time-to-collision. Trials with *Z*-scores greater than +3 or less than −3 were considered outliers and excluded from analyses (1% of trials).

Table 1 shows the ratings for the modified version of the Fear of Spiders Questionnaire (Szymanski and O’Donohue 1995). Mean fear ratings were higher for snakes and spiders than for butterflies and rabbits *t*(29) = 7.88, *p* < 0.0001, *d* = 1.42. This provides a check on our manipulation of how threatening the different types of stimuli were. Table 2 shows the ratings for the Claustrophobia Questionnaire (CLQ; Rachman and Taylor 1993; Radomsky et al. 2001).

**Table 1:** The mean (with SD) fear ratings for the four stimulus categories of the modified version of the Fear of Spiders Questionnaire (Szymanski and O’Donohue 1995) where a score of +3 indicates high levels of fear and −3 indicates low levels of fear

Stimulus category	Mean (SD)
Snakes	0.60 (1.09)
Spiders	−1.11 (0.96)
Butterflies	−2.50 (1.66)
Rabbits	−2.44 (1.82)

**Table 2:** The mean (with SD) of the total mean scores for claustrophobic fear (CLQ total score), for the fear of suffocation subscale (SS) and the fear of restriction subscale (RS)

	Mean (SD)
CLQ total score	60.20 (19.01)
SS subscale	26.56 (8.79)
RS subscale	33.63 (11.72)

**Fig. 1:**
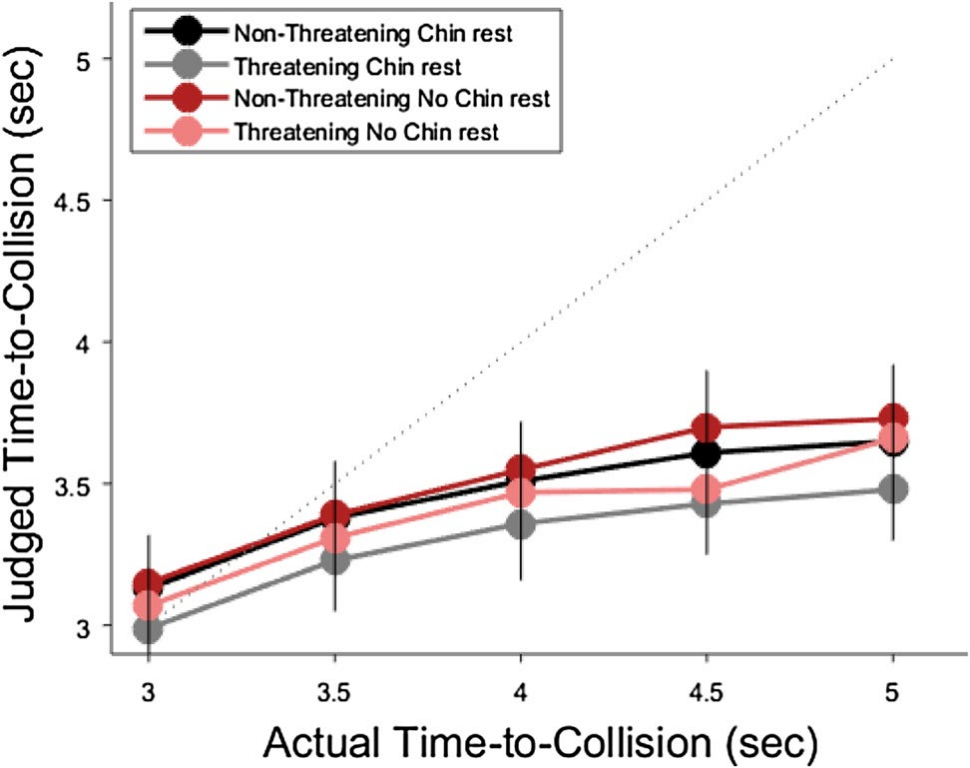
Judged TTC as a function of actual TTC in all the different conditions. Judgments increased monotonically as a function of actual TTC for non-threatening (butterflies and rabbits) and threatening (snakes and spiders) stimuli. There was a clear bias to underestimate TTC for threatening compared to non-threatening stimuli (*grey* and *pink lines*). Moreover, the *black* and *grey lines* appear slightly below the *red* and *pink* ones suggesting that the judgments were reduced in the chin rest condition relative to no chin rest condition. The *grey dotted line* indicates veridical judgments

Figure 1 shows the results. An analysis of variance (ANOVA) was run on mean time-to-collision judgments including the restriction manipulation (chin rest, no chin rest), stimulus category (threatening, non-threatening) and actual time-to-collision (3.0, 3.5, 4.0, 4.5, 5.0 s) as within-subjects factors. There was a significant effect of actual TTC, *F*(4, 116) = 43.56, *p* < 0.0001, $\eta_{\text{p}}^{2}$ = 0.71, with judgments increasing monotonically with actual time-to-collision. There was also a main effect of stimulus category, *F*(1, 29) = 9.98, *p* < 0.005, $\eta_{\text{p}}^{2}$ = 0.26, with judgments being reduced for threatening compared to non-threatening stimuli, a replication of our previous results (Vagnoni et al. 2012, 2015). Finally, there was also a significant effect of the restriction manipulation *F*(1, 29) = 6.05, *p* < 0.03, $\eta_{\text{p}}^{2}$  = 0.17, with judgments being reduced in the chin rest condition relative to no chin rest condition. There were no significant interactions (*p*s > 0.1).

**Fig. 2:**
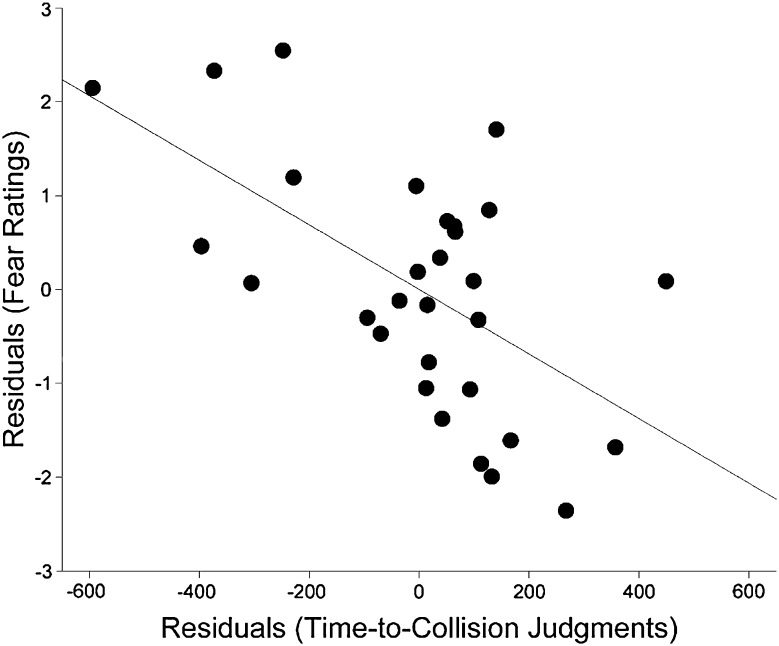
Scatterplot showing relation of TTC judgments and fear. For both TTC judgments and fear ratings, variance specifically related to the threatening stimuli was isolated by calculating the residuals regressing scores for threatening on those for non-threatening stimuli. These residuals were significantly negatively correlated, indicating that greater fear was associated with increased tendency to underestimate TTC. The *grey line* represents the least-squares regression line, regressing fear on TTC judgments

To isolate variance specifically related to individual differences in fear of the threatening stimuli, fear ratings for snakes and spiders were regressed on ratings for butterflies and rabbits, and we calculated the residuals. Similarly, for TTC judgments, mean judgments for threatening stimuli were regressed on judgments for non-threatening stimuli and we calculated the residuals. The residuals estimated how much more afraid of snakes and spiders a participant was than would have been predicted by their fear of butterflies and rabbits. In the case of TTC judgments, the residuals estimated how much earlier a participant judged the arrival time of threatening stimuli than would have been predicted by their TTC for non-threatening stimuli. The residuals for fear and TTC judgments were significantly negatively correlated, *r*(28) = −0.589, *p* < 0.001 (Fig. 2), indicating that people who reported more fear of snakes and spiders, relative to their fear of butterflies and rabbits, showed larger underestimation of TTC of these threatening stimuli. These results replicate the relation between underestimation of TTC and the specific fears of participants we have reported previously (Vagnoni et al. 2012, 2015).

To calculate the correlation between the underestimation effect in the chin rest condition and claustrophobic fear, we regressed TTC judgments in the chin rest condition on those in the no chin rest condition, and calculated the residuals. We then correlated that index with the total scores at the CLQ and separately for the two scales (SS and RS). There was no correlation with the total scores, *r*(28) = 0.13, *p* = 0.46, nor with either the SS scale *r*(28) = 0.06, *p* = 0.38, or with the RS scale *r*(28) = 0.17, *p* = 0.18.

#### Analysis of participant’s views on experimental hypotheses

One potential concern about these results is that the experimental hypotheses may have been transparent to the participants, which may have influenced their performance. On the one hand, we believe that the chin rest is not an obvious manipulation given that it is not commonly used to restrict people and that participants, to comply with our hypotheses, should have made many assumptions (they would have had to have guessed that we were expecting an underestimation of approaching stimuli, that we interpret this underestimation as an adaptive response that allows the observer to have more time to engage in a defensive behaviour, that the chin rest was used to encumber them and that while encumbered they would have underestimated more the TTC). Moreover, if the hypotheses were transparent to the participants we should have found an interaction between chin rest condition and threat as hypothesized but not found.

Nevertheless, given the importance of this point we decided to collect data to address it. To directly investigate this issue, we tested ten participants on an abbreviated version of Experiment 1 and then asked them about their beliefs regarding the experimental hypotheses. Specifically, after completing the behavioural task, we asked participants the following questions and recorded their responses:

“Do you have any thoughts about our experimental hypotheses?”

“Do you think that the chin rest has influenced your responses? How?”

“Do you think that the semantic content of the stimuli has influenced your responses? How?”

The majority of the participants (8 out of 10) did not have any thoughts about our experimental hypotheses or had a wrong guess. Two participants mentioned that they thought that we expected to find faster responses to threatening stimuli. Critically, none of the participants mentioned either that the chin rest led to faster judgments or that they thought that we were expecting such a pattern. When participants described how the chin rest affected their TTC judgments their responses did not follow a specific pattern. Indeed one participant claimed that the stimuli looked less dangerous and further away in the chin rest condition while others, in contrast, felt that the stimuli looked closer when using the chin rest. Moreover, several participants stated that they felt that the chin rest speeded their responses, while others felt that it slowed them, given that the chin rest acted as a distractor or because it had a calming effect.

Neither the descriptions of the effect of the semantic content of the stimuli on the TTC judgments were consistent. Indeed, while some participants had the impression that the threatening stimuli speeded their responses others claimed that they were slowed down by them. Some of the participants reported that it was not the semantic content of the stimuli that influenced their responses but their orientation on the screen (e.g. facing forward/backward) or their size in the real world. Therefore, from these results, it seems that both the restriction effect and the threat effect are not due to the experimental demand characteristics.

### Discussion

We replicated the classic TTC underestimation effect with judged TTC being underestimated relative to actual TTC (McLeod and Ross 1983; Schiff and Oldak 1990; Neuhoff 2001). The underestimation was not present for the shortest TTC, a result consistent with previous findings (McLeod and Ross 1983; Cavallo and Laurent 1988; Schiff et aI. 1992) showing that TTC estimation improves as velocity increases. Sidaway et al. (1996) suggested that more intense optic flow fields increase the accuracy of the perception of TTC. Indeed, a higher velocity of approach means a greater rate of flow in the optic array. Therefore, the rate, and consequently the number, of texture elements crossing the retina increases as velocity of approach increases.

We found that time-to-collision judgments of approaching stimuli were influenced by our motor abilities. Having restricted ability to move led observers to use a more conservative margin of safety. We also replicated our finding that the arrival time of threatening stimuli is underestimated compared to non-threatening stimuli (Vagnoni et al. 2012). However, these two effects did not interact. Restriction of movement produced an overall decrease in judged TTC, but did not modulate the effect of threatening semantic content. It seems that being encumbered led the participants to be more conservative in their judgments with any object approaching their body, such that even if the looming stimulus was represented by a non-threatening object, participants assured themselves with a bigger margin of safety. Importantly, both the effect of restriction and the threat effect do not seem to be mere consequences of the experimental demand characteristics.

Moreover, we replicated our finding that more fearful participants underestimated more the arrival time of the feared objects. However, more claustrophobic participants did not underestimate more the arrival time of looming stimuli when encumbered.

The stronger underestimation of looming stimuli while encumbered is in line with a previous study on looming sounds (Neuhoff et al. 2012). Neuhoff and colleagues (2012) measured each participant’s strength and cardiovascular fitness, demonstrating that listeners with lower levels of strength and cardiovascular fitness have a larger anticipatory bias in their perceived auditory arrival time. In this experiment, we wanted to investigate if transitory changes in the possibility to move also modulate the anticipatory bias.

The adaptive meaning of this effect is clear, having reduced motor abilities leads the observer to use a more conservative margin of safety. Having weaker motor abilities led the observer to underestimate more the arrival time of looming objects. Indeed, the observer that has reduced ability to move needs more time to engage in a defensive behaviour.

Another possibility is that in the chin rest condition participants were wobbling less. It has been shown that adding cross modal noise through wobbling in subject’s position can improve TTC judgments (Ranjit et al. 2015). Several studies have shown how wobbling enhances visual perception. For example, in humans, Repperger et al. (2005) showed that target tracking task can be improved by adding lateral noise to the chair where subjects sat during the task. Necker (2007) argued that head-bobbing (i.e. a rhythmic forward and backward movement of head while walking) enhances depth perception.

## Experiment 2: How general is the modulation of momentary action capability?

One possible hypothesis of the increased underestimation of time-to-collision when observers are restrained is that their PPS representation enlarged during the restriction condition. Having reduced movement ability could influence the representation of our safety zone, leading one to consider an object relatively far as closer because of the awareness of being encumbered. Several tasks have been used to investigate PPS. In this second experiment we employed a behavioural task in which the participants bisect lines at different distances. Previous results have shown that people show a small leftward bias (*pseudoneglect*) when bisecting lines in near space which gradually shifts to a rightward bias with increased viewing distance (e.g. Varnava et al. 2002; Longo and Lourenco 2006, 2007). In this experiment, we asked participants to bisect lines with a laser pointer at several viewing distances with or without a chin rest. Here, we investigated the generality of the effect of the chin rest found in Experiment 1. If there is a modulation of the capability of movement in this task the chin rest manipulation should affect the spatial gradient of bisection biases, leading to a reduction in rightward bias at farther distances. Previous work has shown that increasing effort on a line bisection task leads to a contraction of PPS (Lourenco and Longo 2009). Specifically, participants wearing heavy weights on their wrist showed more rightward bias at the closest distances, and a more gradual rightward shift with increasing distance, suggesting that the nearest locations were represented as being farther away (Lourenco and Longo 2009).

### Method

#### Participants

Nineteen members of the Birkbeck community (nine female) between 22 and 50 years of age, mean age 34.4 years, participated for payment or course credit. The sample size in this experiment was smaller relative to Experiment 1 given that there were fewer experimental conditions. Participants were generally right-handed as assessed by the Edinburgh Inventory (M: 72.98, range −90.9 to 100) (Oldfield 1971). Participants reported normal or corrected-to-normal vision. Procedures were approved by the local ethics committee.

#### Stimuli, design, and procedure

Stimuli were lines (height: 1 mm) of 4 different lengths, from 4 cm (length 1), 8 cm (length 2), 16 cm (length 3) to 32 cm (length 4). The lines were grey and drawn on a laminated matte black A3 paper (width 29.7 cm, height 42 cm).

Participants were tested in a large room where they bisected lines of 4, 8, 16, and 32 cm using a laser pointer at six distances from 30 to 180 cm, at 30-cm intervals (30, 60, 90, 120, 150, and 180 cm). Distances were marked on the floor with tape. Lines were centred on A3 laminated matte paper and attached horizontally to a whiteboard. A different sheet of paper was attached to the whiteboard on each trial. A laser pointer was continuously activated and attached to the head of a tripod, the height of which was adjusted for each participant’s comfort. The tripod was positioned to the right of the participant. When the participant thought to have found the midpoint of the line, a picture of the pointing was taken with a webcam controlled through a MATLAB (Mathworks, Natick, MA) script.

There were a total of 96 trials, during half of them the participants were performing the bisection using the chin rest (chin rest condition) while in the other half they were performing the bisection without the chin rest (no chin rest condition). The conditions were pseudorandomized in an ABBA order.

#### Analysis

Data from two participants were removed due to computer problems. For each participant, mean percent deviations were calculated for each distance and for each condition (chin rest vs. no chin rest). In each condition, data were fit with multiple linear regression for each participant, and parameter estimates of slope and y-intercepts were used for subsequent analyses. The slope in the analysis indexes the rate at which bias shifts rightward with increasing distance, a measure of the ‘‘size” of PPS. It has been previously shown that tool use produces a reduction of slope without a corresponding change in intercept (Longo and Lourenco 2006; Hunley et al. 2017), indicating that closer and farther distances become less distinct with the farther distances being treated as if they are nearer in space—an extension of PPS. Moreover, we tested for a reduction of slope in the chin rest condition given that we hypothesized that the use of the chin rest would have expanded the PPS of the participants.

### Results and discussion

**Fig. 3:**
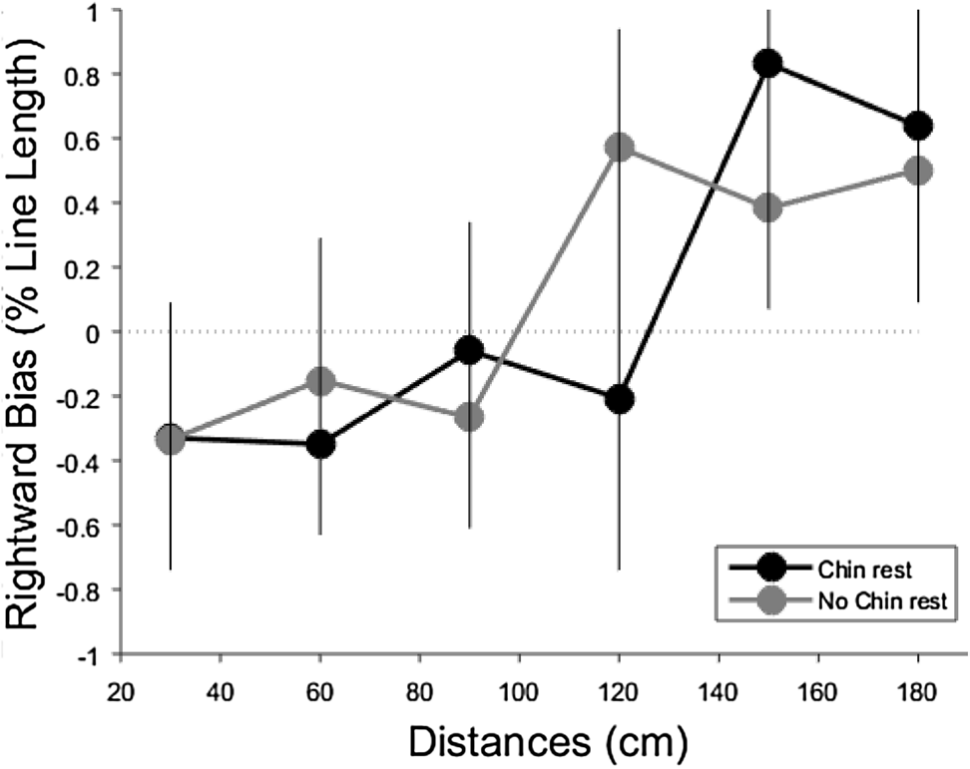
Mean (and SE) rightward bisection bias for chin rest and no chin rest conditions. Negative values indicate leftward bias while positive values rightward bias

We found significant rightward shifts in bias with increasing viewing distance in both the chin rest condition (mean slope, *β* = 0.73 line length/meter), *t*(18) = 3.15, *p* < 0.01, *d* = 0.69, and no chin rest condition (mean slope, *β* = 0.63 line length/meter), *t*(18) = 2.58, *p* < 0.02, *d* = 0.56. This replicates previous reports that viewing distance modules bisection biases (e.g. Varnava et al. 2002; Longo and Lourenco 2006, 2007). Critically, however, this shift was not modulated by the chin rest and was similar in both conditions, *t*(18) = 0.39 *p* = 0.70, *d*_*z*_ = 0.08 (Fig. 3). Further, the mean *y*-intercept was not significantly different in the two conditions *t*(18) = −0.44, *p* = 0.66, *d* = 0.09.

Data were also analysed using analysis of variance (ANOVA) with condition (chin rest vs. no chin rest) and distance (30–180 cm) as within-subjects factors. There was a significant main effect of distance, *F*(5, 80) = 4.61, *p* = 0.001, $\eta_{\text{p}}^{2}$ = 0.22, but, critically, no effect of condition, *F*(1, 16) = 0.00, *p* = 0.96, $\eta_{\text{p}}^{2}$ = 0.00, nor an interaction between distance and condition, *F*(5, 80) = 1.73, *p* = 0.13, $\eta_{\text{p}}^{2}$  = 0.09, suggesting that the rightward shift in bias across distance was not affected by the chin rest manipulation (Fig. 3).

These results provide a clear replication of the shift in lateral attentional bias between near space and far space. Critically, however, this transition does not appear to be modulated by the use of a chin rest. To exclude the possibility that the lack of the immobilization effect in Experiment 2 is not merely linked to lack of power (smaller sample size relative to Experiment 1) we performed a Bayesian Repeated Measures ANOVA using JASP software. The results show that the Bayes Factor of the null hypothesis against the alternative hypothesis (BF 01) for the factor chin rest, no chin rest is equal to 6.610, which constitutes moderate evidence in support of the null hypothesis. Therefore, it seems that the non-significant results of the chin rest manipulation are not due to lack of power. In contrast, the BF 01 for the factor distances is 0.008, which represents strong evidence for the alternative hypothesis. This means that, contrary to the effect of chin rest, the effect of distance can be considered a strong effect.

There are several possible explanations for the lack of the immobilization effect in Experiment 2. It is possible that the effect found in the first experiment is not linked directly to representations of PPS. In this case, the immobilization effect could be described as an anticipatory bias modulated by the motor system’s capability.

Another possibility is that the line bisection task has been used to investigate the “space for action” function of the PPS. It is conceivable that the chin rest manipulation does not influence the “space for action” but only the “defensive” function of the PPS (de Vignemont and Iannetti 2015). Alternatively, immobilization with a chin rest may have effects specific to head-centred PPS. Lourenco and Longo (2009) found that bisection biases were clearly modulated by applying heavy wrist weights to the arm. No such effect, however, was found for wearing a heavy backpack, though that manipulation has been shown to modulate other processes, such as distance perception (Proffitt et al. 2003) and locomotor imagery (Decety et al. 1989). Thus, the bisection task may specifically reflect hand-centred peripersonal space and, different methods of movement restriction (heavy backpack, wrist weights, chin rest) may produce selective effects on different aspects of perception. Another possibility is represented by the fact that visual-motor calibration depends on response modality (Kunz et al. 2013). Indeed, it has been found that there is no transfer of visuo-motor experience/calibration between two different response methods.

## General discussion

Our motor abilities influence TTC judgments. Indeed, having less possibility of movement leads observers to adjust their judgments so that they assure themselves with a margin of time to start a defensive response if needed. In Experiment 1, participants showed reduced TTC judgments when they were immobilized with a chin rest compared to when they stood freely. The results also provided a clear replication of our previous finding that TTC judgments of threatening stimuli (i.e. snakes, spiders) are underestimated compared to non-threatening stimuli (i.e. butterflies, rabbits) and that this effect is correlated with participant’s specific fears of these categories (Vagnoni et al. 2012, 2015). These two effects, however, did not interact as hypothesized. Indeed, immobilization did not modulate the effect of threat. Experiencing the immobilization could have enhanced the margin of safety independently from the semantic content of the approaching object so that participants were more conservative in their judgments even when the looming object was represented by a non-threatening stimulus.

This immobilization effect is consistent with a recent finding (Kandula et al. 2016) where less skilled participants (non-video games players) underestimated the location of an approaching ball only when the task was difficult; indeed, the momentary action capability, in this case, was manipulated using two difficulty levels in a virtual reality experiment. The underestimation of looming stimuli in the encumbered condition is not only in line with previous ones showing how physical fitness influences TTC judgments (Neuhoff et al. 2012) but it also extends them. Indeed, we recalibrate our judgments even for temporary changes in our motor abilities. This result has clear adaptive meaning: if our body is temporarily encumbered, then we need more time to engage in a defensive behaviour. Once again, we showed that threatening stimuli were more underestimated than non-threatening stimuli and that more fearful participants underestimated more the arrival time of the feared objects. These effects could have an evolutionary origin. It has been proposed that fear shaped our visual system (Isbell 2009). In particular, it has been argued that the fear of snakes prompted the evolutionary changes in the visual system of mammals (Isbell 2009). However, it is interesting to note that the same was not true for claustrophobic fear: observers who were more claustrophobic did not underestimate more the arrival time of looming stimuli in the chin rest condition. Moreover, we found no interaction between the two main effects, so that immobilization did not modulate the effect of threat.

Both our motor abilities and individual differences modulate TTC judgments. However, we do not know at which stage of information processing this modulation occurs. Several authors claim that emotion, internal states, and physical efforts influence perception, making people aware of both the opportunities and the costs associated with action (Proffitt 2006). Another possibility is that the recalibration intervenes at later post-perceptual stages (Firestone 2013; Firestone and Scholl 2014), for example, during action planning. On this interpretation, the perceived rate of expansion on the retina would be interpreted accurately, while judgments would be influenced by the semantic content of the stimulus, our specific fears, and our promptness to react. Thus, although our results provide evidence that restricted action ability influences TTC judgments, they do not provide clear evidence about whether this modulation occurs at a perceptual level, or at later cognitive stages in which the results of perceptual processing are used to construct a behavioural judgment. It is important to note that from our results it seems that both the effect of immobilization and the threat effects are not simply due to the experimental demand characteristics.

Looming stimuli have been used in different paradigms to investigate PPS representation both in monkeys (Cooke and Graziano 2003; Graziano and Cooke 2006) and humans (Canzoneri et al. 2012). The effects found with the TTC task could be interpreted as changes in PPS. For example, the underestimation of TTC could be interpreted as an enlargement of PPS. Previous studies have shown how PPS is flexible, so that the possibility to act in the far space through a tool expands the PPS (Berti and Frassinetti 2000) while the effort in performing an action shrinks it (Lourenco and Longo 2009). In our case, being mildly encumbered would have enlarged our PPS so that stimuli are considered entering the PPS earlier in the encumbered condition relative to when the participants were not encumbered.

With Experiment 2, we were specifically interested in investigating the generality of the modulation of momentary action ability. The results of Experiment 2 showed no effect of the chin rest manipulation. It is possible that the immobilization effect found in the first experiment does not represent a consequence of the PPS expansion but an anticipatory bias modulated by the motor system’s capability. However, another possibility is that in the two experiments we are examining two different aspects of PPS. Recently, a dual model of peripersonal space has been proposed, based on a clear functional distinction between bodily protection and goal-directed action (de Vignemont and Iannetti 2015). The authors argue that the two functions of PPS require distinct sensory and motor processes that obey different principles. It is possible that in the first experiment we explored the “defensive” function of the peripersonal space whereas in the second experiment we explored the “space for action” function. It is true that the line bisection task has been widely used to investigate the modulation of the PPS intended as the “reaching space” and in the task used the participants were always capable of bisecting the line, the chin rest did not impair their possibility of action on the environment. An explanation for the lack of the chin rest effect in the Experiment 2 is that this manipulation modulates the defensive PPS while failing to modulate the “space for action” PPS. Indeed, even if mildly encumbered, participants were always able to bisect the lines, such that there were no significant consequences on their actions.
